# Building a house on shifting sand: methodological considerations when evaluating the implementation and adoption of national electronic health record systems

**DOI:** 10.1186/1472-6963-12-105

**Published:** 2012-04-30

**Authors:** Amirhossein Takian, Dimitra Petrakaki, Tony Cornford, Aziz Sheikh, Nicholas Barber

**Affiliations:** 1 Division of Health Studies, School of Health Sciences & Social Care, Brunel University, Uxbridge, UB8 3PH, UK; 2 Department of Business and Management, School of Business, Management & Economics, University of Sussex, Brighton, BN1 9QF, UK; 3 Department of Management, London School of Economics & Political Science, London, WC2A 2AE, UK; 4 eHealth Research Group, Centre for Population Health Sciences, The University of Edinburgh, Edinburgh, EH8 9DX, UK; 5 Department of Practice and Policy, UCL School of Pharmacy, London, WC1H 9JP, , UK

**Keywords:** Electronic health record (EHR), Evaluation, Methodology, Sociotechnical, Changing, NHS CRS, Adaptation, Reflexivity

## Abstract

****Background**:**

A commitment to Electronic Health Record (EHR) systems now constitutes a core part of many governments’ healthcare reform strategies. The resulting politically-initiated large-scale or national EHR endeavors are challenging because of their ambitious agendas of change, the scale of resources needed to make them work, the (relatively) short timescales set, and the large number of stakeholders involved, all of whom pursue somewhat different interests. These initiatives need to be evaluated to establish if they improve care and represent value for money.

****Methods**:**

Critical reflections on these complexities in the light of experience of undertaking the first national, longitudinal, and sociotechnical evaluation of the implementation and adoption of England’s National Health Service’s Care Records Service (NHS CRS).

****Results/discussion**:**

We advance two key arguments. First, national programs for EHR implementations are likely to take place in the shifting sands of evolving sociopolitical and sociotechnical and contexts, which are likely to shape them in significant ways. This poses challenges to conventional evaluation approaches which draw on a model of baseline operations → intervention → changed operations (outcome). Second, evaluation of such programs must account for this changing context by adapting to it. This requires careful and creative choice of ontological, epistemological and methodological assumptions.

****Summary**:**

New and significant challenges are faced in evaluating national EHR implementation endeavors. Based on experiences from this national evaluation of the implementation and adoption of the NHS CRS in England, we argue for an approach to these evaluations which moves away from seeing EHR systems as Information and Communication Technologies (ICT) projects requiring an essentially outcome-centred assessment towards a more interpretive approach that reflects the situated and evolving nature of EHR seen within multiple specific settings and reflecting a constantly changing milieu of policies, strategies and software, with constant interactions across such boundaries.

## **Background**

Justification for substantial investments in Electronic Health Record (EHR)^1^ systems and infrastructure, as seen in many national healthcare strategies, comes from the promise that EHR will make healthcare better, safer, cheaper, more efficient and more integrated. All this is to be achieved through, amongst other things, improved availability, completeness and legibility of patient records, standardization of care practices and more informed and timely clinical decision making [1-5]. The evidence basis of such claims needs to be made clear, not just assumed [6]. Hence evaluation studies aim to test their validity and trace the achievement of specific outcomes [7]. Evaluation also serves associated goals including legitimizing investments, informing and educating key constituencies, and providing an ever-stronger evidence base for policy makers [8-12].

Most EHR evaluations draw upon a broadly positivist ontology and pursue causality in term of an intervention’s impact and by making objective judgments concerning the outcomes and hence degree of success or failure of such initiatives (see for example the studies of EHR summarized in references 8 and 11) [8,11]. This type of evaluation draws upon and reflects the principles of science in general and evidence-based medicine (EBM) in particular. Study designs include systematic reviews [13]; descriptive theory-based case studies [13-15]; ‘observational’; ‘quasi-experimental’ [11,16-18]; before-after, and, less frequently, randomized controlled trials (RCTs) [19].

Each of these designs has strengths and weaknesses, but if pursued in isolation runs the risk of over-simplifying the dynamic complexity of large-scale technology-led projects such as national EHR initiatives. The argument presented in this paper is that the issues of context found in large-scale projects cannot be ‘controlled’ by traditional research design alone, but have to be embraced and actively incorporated into evaluation. We describe how we met this methodological challenge in our work allowing us to incorporate and reflect a changing sociopolitical and sociotechnical context. We conclude from this experience that conducting meaningful research in a dynamic environment requires methodological reflection and adaptation. Thus we see national EHR endeavors not as programs composed of essentially discrete ICT ‘projects’, dissociated from policy, technology, service delivery and clinical work. Rather, we see the need to incorporate these elements in evaluation as inextricable parts of EHR programs, including the constantly changing parallel policies and strategies, complex and evolving software ecologies and diverse health care working practices, all of which interact across their porous boundaries [20].

We are not the first to suggest the importance of recognizing the multi-dimensional, contextual and sociotechnical character of health care information systems seen within complex adaptive health care environments [see for example 15, [21-25]. Nevertheless, much literature on the evaluation of EHR implementations has been narrowly focused on individual hospital or clinical settings [26], or in a limited number of hospital sites as they embark on relatively small-scale, home-grown or discrete EHR initiatives [27]. This is unsurprising given that only limited national-scale implementations of EHR systems have taken place to date, and there is as a consequence little published evidence to inform larger scale evaluations [28]. Indeed, choice of appropriate methods for such evaluations remains a contested issue [29,30]. The contribution we make to this debate, drawing from our own experience, emphasizes two principal elements. First, we recognize the malleable character of any EHR program as it is shaped by contextual forces and is reinterpreted by various interest groups and people. Second, we take from this the need for evaluators to draw upon alternative perspectives and understandings of technology and the possible role of information and data in changing work practices and organizational structures and hence potential to effect specific outcomes. These two fundamental ideas are both ontological (i.e. concerned with the assumptions made as to the nature of the reality we study) and epistemological (i.e. concerned with how we obtain valid information about that reality)[9].

### **Background to the English EHR initiative: the NHS CRS**

Much has and will be written about the England’s National Programme for Information Technology (NPfIT), the government agency delivering it, NHS Connecting for Health (NHS CFH) and the EHR element at its centre: the NHS Care Records Service (NHS CRS). We do not rehearse at length this history here, but rather seek to frame and contextualize the evaluation task we undertook and from which we have derived the findings presented here.

In 2002, the UK Department of Health (DH) chose to procure and implement on a national scale a limited number of commercial software packages to provide EHR capacity for secondary care in England. This was to be delivered (e.g. implemented and supported) in National Health Service (NHS) hospitals by a small number of centrally contracted Local Service Providers (LSPs) each working within a geographical region [31,32]. The LSPs were major corporations in the ICT services sector, contracted to deliver standard software systems to local NHS organizations, ensuring system integration, interoperability and national connectivity [33]. The overall $19.6 (£12.7) billion program was conceived as a strategic initiative to move the English NHS towards an integrated set of electronic systems and data infrastructures that would transform healthcare [28,34]. The EHR element of the NPfIT – NHS CRS – was from the outset regarded as the core deliverable [35]. The intention was that new software systems installed in individual NHS Trusts^2^ would connect to national databases and a messaging service (the ‘NHS Spine’). The NHS CRS would then be in two parts: a centrally stored summary care record (SCR) drawing principally from primary care and containing basic clinical information for emergencies [36,37], and a locally held and shared detailed care record (DCR). The latter was the focus of our research.

**Figure 1:**
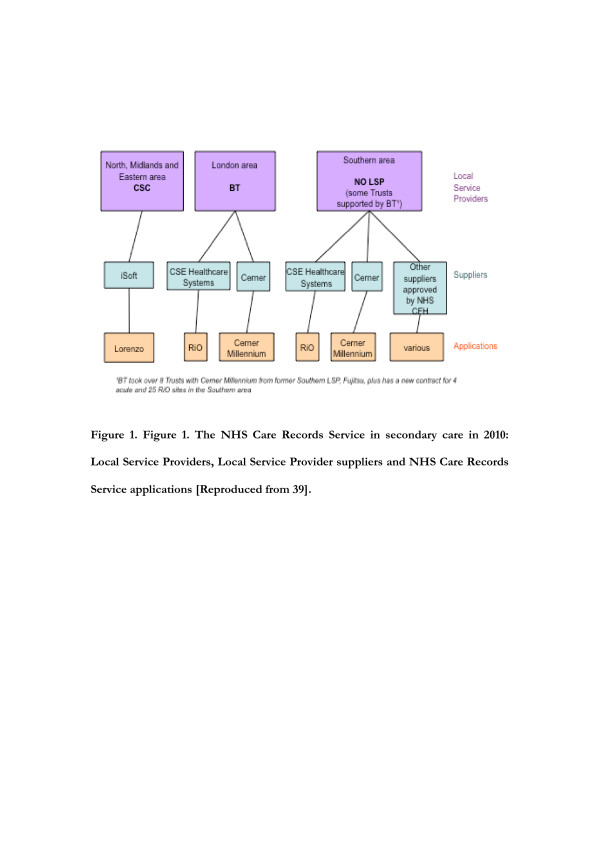
The NHS Care Records Service in secondary care in 2010: Local Service Providers, Local Service Provider suppliers and NHS Care Records Service applications [Reproduced from 39].

At the outset (2004), the NHS CRS was to be delivered within five geographically based implementation regions (also known as clusters) by separate LSPs. The five implementation clusters were subsequently reduced to four by the merger of two clusters in the bidding process (2005) and subsequently to three by the departure of one contracted LSP (2006). Another LSP departed in 2008 when it failed to renegotiate its contract, leaving only two LSPs active (Computer Sciences Corporation: CSC & British Telecom: BT) serving the three remaining geographical clusters: the North, Midlands and East (NME), London and the some parts of the South [34]. At the outset, each cluster had a phased plan for EHR that proposed delivery of incremental functionality through a sequence of software releases. LSPs for the London and Southern cluster planned to deploy sequential releases of Cerner’s Millennium software for acute Trusts and CSE International’s RiO software for mental health and community services, while the NME area planned sequential releases of iSOFT Lorenzo software (see Figure 1).

At the time of its selection (2006), Millennium was a well-established ‘off the shelf’ EHR software in use in the USA and elsewhere, including in one hospital in England. RiO was also a commercial ‘off the shelf’ software, but this had been developed in the UK for over 10 years for mental health and community Trusts. Finally, iSoft’s Lorenzo was a ‘home-grown’ product, being developed in line with requirements and specifications of English NHS Trusts. As an evolving product, the implementation of Lorenzo in the NME cluster was gradual and at first occurred on a small scale and at a relatively slow pace. It was implemented in parts of clinical departments, wards or pathways in a limited number of Trusts (this was referred to as a ‘soft landing’). By contrast, Millennium and RiO implementations followed ‘big bang’ Trust-wide approach.

### **The initial design of the NHS CRS evaluation study**

The successful implementation of the NHS CRS was seen as a critical factor for the success of the NPfIT [38]. An independent evaluation programme was established in 2006 – the CFH Evaluation Programme (CFHEP)^3^ – to mirror the range of activities of NHS CFH in delivering the various parts of the NPfIT [28]. Our team was commissioned to undertake a 30-month (September 2008-March 2011) formative and summative evaluation of the implementation and adoption of NHS CRS in hospitals in England. We conducted this as a real-time evaluation undertaken as EHR systems were actively being implemented and used in healthcare settings. Our findings have been reported elsewhere [39,40].

The initial research plan was based on the ability to track developments over time in a number of NHS Trusts, undertaking a before-during-after assessment. This approach was in part dictated by the commissioning brief, which emphasized before-after comparative work, measurements of the ‘impact’ of EHR systems, and the need to obtain insights into the costs of deployment and cost-effectiveness. But from the outset we were convinced that the process of delivery of the systems – their implementation and adoption, the ‘during’ – was significant. The original theoretical approach was also informed in part by a ‘realistic evaluation’ perspective [41], as also advocated by the commissioning brief, posing the question: “Which aspects of the NHS CRS work for whom and under what circumstances?”

Thus, from the outset we recognized EHR systems as implying complex processes of change driven not only by the new technologies, but also by many human-related, managerial, organizational, institutional and cultural discourses [9,11,42,43]. We therefore chose to undertake our work framed within Cornford et al.’s (1994) socio-technical evaluation framework which is presented as a matrix of Donabedian’s concepts of structure, process and outcome set against dimensions of system functions, human perspectives and organizational settings (See Table 1) [44]. This framework was used to shape and frame both data collection and analysis.

**Table 1:** **The sociotechnical model used in framing the study **[[44]]

	**System Functions**	**Human Perspectives**	**The Health Care System**
**Structure**	Technology	Work Organization	Role of Medicines
What has been put in place?	What does the intervention look like?	What new work practices emerge?	What is the role of the intervention in the wider healthcare system?
**Process**	Processing	Social Interactions	Management of Care Delivery
What is done that is different?	What processes emerge and change?	How do communication patterns and workflow change?	What organizational changes emerge?
**Outcome**	Validity of Processing	Quality of The Service	Potential for Change
What has been achieved?	Is the service safe and reliable?	Is the experience of the service satisfactory?	How might the intervention be used in the future?

Our evaluation focused on the NHS CRS and the software that embodied it as it arrived in contexts rich with existing structures and resources, and explored how this was accommodated and then integrated (or not) in the work processes of the specific NHS Trusts studied. We assumed from the outset that this would lead to diverse outcomes that reflected these distinct contexts.

The scope of the research included all stages of implementation and adoption from initial awareness and planning through to sustained use. The sites identified for detailed study were hospitals and other secondary care and community care Trusts across England that were (or would be) implementing NHS CRS applications in the early phases of the national roll-out, so-called ‘early adopters’. Our evaluation explored the implementation processes across Trusts by looking into the organizational activities undertaken and tracing the consequences for professional roles, workflows and clinical practices, and opportunities found for organizational learning. This suggested attention to the expectations and attitudes of key stakeholders, and policy-related aspects of implementation and adoption. In addition, we investigated the availability of clinical notes in outpatient clinics and assessed, as best we could, the costs of implementation at the Trust level.

Participating hospitals were selected according to their projected implementation timelines, initially based on the principles of a stepped-wedge design [45]. The plan was to recruit up to five ‘early adopter’ sites in each of the three implementation clusters and to purposively include a range of representative Trusts (i.e. teaching, non-teaching, foundation, acute & mental health, and district hospitals) to allow comparisons.

This approach had limitations. The NHS CRS was initially planned to be delivered in each region as four main software releases, over a period of three or more years. This meant that this evaluation could reflect only a part of the implementation process and might miss significant longer-term organizational changes, given the relatively short-term nature of the study (i.e. 30 months). Further, the three key NHS CRS software systems studied – Millennium, RiO and Lorenzo – would provide different mixes of functionalities in different releases and for different sites, rendering comparisons between sites, regions or software difficult.

More critically, our research plans were predicated on the belief that there would be clearly distinguishable periods defined by the absence and then presence of NHS CRS in hospitals. This would allow us to make comparisons based on before (absence), during (implementation) and after (presence). Although these periods would be different due to varied implementation strategies (e.g. sites implemented systems with different functionalities in different sequence following different implementation plans), they would nonetheless be broadly comparable in other respects (e.g., approach to training, changeover strategy, resources available, objectives set), thereby allowing comparisons to be made across sites on the basis of the process of implementation and adoption.

### **Shifting sands: key challenges in the implementation and adoption of the NHS CRS delivery**

Some of the assumptions and strategies outlined above were brought into question as soon as our fieldwork started. We outline below the key challenges we encountered and which demanded reflexive adaptation in the research approach.

#### ***‘Translating’ the NHS CRS: from single to multiple understandings and strategies***

The introduction of NHS CRS as an idea, let alone an operational software, into early adopter hospitals proved more challenging than initially anticipated. Although the NHS CRS started from a single vision of a nationally shared EHR, this vision was translated into multiple local visions. This in turn led to the development of a number of distinct local strategies for its implementation and adoption (or in some cases non-adoption, or even dis-adoption). Whilst progress towards EHR systems was almost universally felt to be important for the NHS, it often became secondary to other, more pressing, local and national priorities. For instance, NHS Trusts that offered services in multiple sites saw the NHS CRS as a means to create shareable electronic records, and to connect the local community services with the hospital, thereby addressing problems of missing records, cross-site working and primary and secondary care integration. Other Trusts looked at it as a chance to update their old IT systems, and others for bringing about Trust-wide and strategic organizational change, facilitating mergers or reshaping their administrative structures. Few saw the national vision as the principal driving force for their own engagement; indeed many found the national vision fundamentally unconvincing.

Delays in delivering systems, different levels of preparedness of ‘early adopters’, and sometimes opposing and conflicting views within NHS Trusts about what the NHS CRS was or should be, resulted in hospitals developing a range of strategies and approaches to their own implementation of EHR systems. Some Trusts revised and postponed their implementation timelines many times in the face of perceived uncertainties. A few Trusts modified the scope of the implementation, whilst others took the more radical decision to opt-out of the NPfIT and pursue their own solutions independently. For example, some hospitals in the London cluster adopted a ‘big bang’ approach to the implementation of the NHS CRS software. Other hospitals with substantial clinical systems in place were reluctant to discard them – a feeling that was reinforced during the period of this study and as they observed the problems faced by others. Hospitals in the NME area were committed to an incremental ‘soft-landing’ approach to implementation, given the limited amount of operational software they were offered. Trusts in the Southern area that received early and basic releases of software had to develop new strategies and seek out their own solutions following the departure of their LSP and the resulting collapse of their ongoing implementation strategy.

In light of the above differences, we reviewed our initial plans. Clearly implementation was highly context-bound. Direct comparisons or summations across the various Trusts experiences and their implementation stages would risk losing much of the valuable local detail if standard measures for comparison were abstracted away from the rich and complex causal environments found in each site. The results would be arbitrary, and certainly would provide limited information as to *how* and *why* EHR implementations progressed as they did.

#### ***Configuration & development of the NHS CRS***

The software packages embodying the NHS CRS were neither self-contained nor did they tend to directly satisfy local requirements. Their introduction was thus accompanied and influenced by a number of interim software solutions and legacy systems with which they had to interact. All software solutions in any case had to be configured (to a greater or lesser extent) in order to meet local purposes and needs [10,46]. In the NME cluster, in particular, implementation team members and clinical staff had to become involved in substantial ongoing activities of development, design and testing of the software in order to tailor the growing software to their (and by implication the wider NHS’s) needs.

Thus, in contrast to our initial assumption that comparisons could be made on the basis of absence and then presence of NHS CRS systems in the hospitals under investigation, in practice we encountered many complex processes concerned not so much with ‘using’ these systems more or less as they were delivered, but of ‘making’ these systems as they were adapted and applied to the work at hand and the goals of the particular Trust.

#### ***Geographical & institutional distribution***

The third challenge we needed to reflect was the distributed ecology of the NHS CRS as it came into being through the intervention of a number of institutions including the Department of Health (DH), NHS CFH (the agency managing the delivery), the LSPs, software providers, hospital and community Trusts, and many other professional, regulatory- and industry-based stakeholders. For example, local implementations of the NHS CRS were undertaken by the implementing hospital, NHS CFH and the LSPs in some form of collaboration and with varying degrees of local site configuration as negotiated by each Trust; decisions concerning the national ‘roll out’ were taken centrally by the DH and NHS CFH; the NHS CRS software products were developed by commercial software developers that were in some cases located outside England (e.g., Lorenzo software was developed in India and Cerner, Millennium’s developer, was based in the USA).

Such distribution posed an important methodological challenge suggesting that the NHS CRS could not be sufficiently understood separately and outside of the organizations that created and shaped it. This insight signified that we could neither capture all stages of the NHS CRS implementation nor always be in the right place at the right time. Rather, we had to accept that we were often present in places where the action was not [47] and thus that the evaluation was inevitably partial (both in the sense of being incomplete and of being biased).

#### ***Political dimensions to the NHS CRS***

One significant part of this complex ecology was its Politics (with both a capital and lower case p). NHS CRS was a political project from the start, constituting an important part of the then [New Labour] government health agenda. It equally was a target of the then opposition, particularly as time lines slipped and delivery stalled. Well before the Labour administration left office in May 2010, the NPfIT came to face significant challenges which led to restructuring of NHS CFH and relevant parts of DH, the exit of two LSPs, ceding of more choice and freedom to Trusts, and revised contractual arrangements. Further, although conceived in a time of relative plenty, by the end of the decade the work to implement NHS CRS was taking place in economically constrained times [39]. This had consequences for how NHS Trusts facing a future of reduced funding made decisions about their commitment to NHS CRS.

Thus as time went on, significant uncertainties about the future of the NHS CRS and its implementation arose [34,48]. Indeed, the new Conservative Liberal-Democrat coalition government’s White Paper (‘Equity and Excellence: Liberating the NHS’ 2010) [49], and their new ICT Strategy consultation [50], proposed major changes in the NHS through structural reorganization with major consequences for EHR initiatives, including greater patient choice (e.g., in control over access and sharing of their electronic records) and greater financial and decisional autonomy to Trusts.

This political nature of the NHS CRS was understood by most stakeholders – often it was what they wanted to talk about most [40] – but this constituted a significant methodological challenge to account for such influences. Rather, these political dimensions added further evidence of the dynamic and malleable character of NHS CRS. This also made it difficult for us as researchers and evaluators, and for research participants, to deconstruct the shifting assumptions upon which NHS CRS was based, and unpack its very particular, centralized, delivery mechanisms, let alone evaluate them. Perhaps most important was the enduring sense of potential for further changes in policies and anticipated funding which led to changes in implementation commitment at the Trust level.

### **Learning methodological reflexivity: adaptation to the shifting sand**

The characteristics described above, although at times more and at other times less visible, were an inextricable part of the NHS CRS we ended up evaluating. Our sense of the protean nature of NHS CRS, its changeability and embodiment within multiple local visions for change, led to a constructive debate within our multidisciplinary evaluation team as we reflexively adapted our evaluation approach**.** Some of the characteristics described above were probably local and contingent, and thus not transferable to other evaluation contexts. Most, however, would be paralleled in any large-scale national EHR program and can serve the development of transferable lessons. In this section we outline the principal insights we gained into evaluation of large-scale EHR programs *,* before explaining them in further detail:

 • EHR programs are inherently political and exist within a dynamic environment

 • EHR is a sociotechnical intervention that is given meaning through the activities of its implementation and adoption. EHR is performed

 • Implementation of EHR systems can be evaluated by studying changing as it occurs, rather than just by measuring achieved change (desired outcomes)

 • Before-after evaluation designs may miss vital information about the change process, and assume that there is a clear definition of ‘after’

· EHR is understood by studying what people do, and in particular how, and to what extent, they ‘work to make it work’

 • Evaluations that are focused on changing narrate the system change and tell the story of EHR implementation and adoption through multiple voices. This is fundamentally distinct from outcome-centric methods that judge EHR as degrees of achievement of an assumed end point, which may take long-time to happen (if it happens at all)

 • Case study-based evaluations can probe deeper and can address EHR systems within dynamic or distinctive socio-cultural environments

 • Multiple coordinated case studies allow an insightful cross-site dialogue that can reveal common themes and distinct experiences

 • The evaluators’ role is to be in part an insider and in part an outsider; understanding but also questioning

• Evaluations in this style can speak of outcomes in terms of concepts such as changed expectations, recognition of benefits, assessments of risk, perceptions of consequence, processes of learning

 • Such findings can be of real use for health services managers and policy makers, and help address pro-innovation bias and techno-centric dreams. It can also facilitate organizational learning

#### ***Processes of change or ‘socio-technical changing’ (ontological lessons)***

Our early experience of NHS CRS implementation led us to step back a little from concerns with outcomes and impact. Our attention turned to creating multiple detailed narratives of the process of change initiated around the NHS CRS. We refer to such change, as it happens as ‘changing’ (present participle) [51]. Conventionally, studying change denotes a movement from one situation to another seen through some comparison of the before and after states. Objective measures of change are sought in such comparisons based on static views or ‘snapshots’ of the context under investigation at two or more locations (e.g. controlled trial) or two or more points in time (e.g. before–after study). Such an approach provides a limited basis upon which to explain the process of change itself (i.e., the internal and ongoing ‘how things change’) and the reasons (i.e., ‘why things change’). Our approach, in contrast, became more and more one that focused on the activity ‘in between’; the period during which things (and people, and teams) were changing, rather than some end state of achieved and stabilized change. This perspective is described in some sociological literature as a performative view; one which sees EHR systems as being brought into existence by the various ways in which actors speak about, act and enact EHR [52]. The EHR thus comes into being as and when it is performed (not when software is delivered and installed) even to the extent that it ‘vanishes when it is no longer performed’ [53].

We developed this performative view by exploring how people acted to make the NHS CRS work, enabled and constrained, as they were by their own skills, attitudes and the various technologies and other resources available to them. We collected views on the implementation as seen by a diverse set of stakeholders [28,54] and sought to explain how their understandings and actions shaped NHS CRS [46]. In this way we were able to understand how the NHS CRS was formed (i.e. how it was ‘per-formed’), translated and reproduced in various sites and settings [55] and the different meanings it embodied for different people, at different times and locations [56].

This led to a subtle, but fundamental shift in the object and purpose of our research; the focus became directed not so much on evaluating a concrete or summative NHS CRS implementation, and thus on making judgments as to what was ultimately achieved, but on understanding and narrating the stories of a network of NHS CRS *in-the-making *[57,58]. In this way we became less concerned to assess progress or achievements measured against predefined criteria, expectations and project milestones. Rather we saw that greater insights could be gained from approaches that sought to ‘tell the whole story’ not just the ending [59].

#### ***Evaluating through interpretation and exploration (epistemological lessons)***

The different local and regional deployment strategies, combined with the limited active deployments at the time of the fieldwork, resulted in a decision to move from comparative evaluation (before-after and between sites) to a linked sequence of case-based studies developing depth of insight into how the multiple NHS CRSs were made to work (to degrees) locally. Under the modified design, individual researchers became deeply immersed in the research sites [30,60]. The shift to a case study-based approach made us, as researchers, responsible for working within the study sites as ‘almost insiders’ or witnesses, required to comprehend what took place in the settings as seen through participants eyes. At other times, researchers were required to be ‘outsiders’ with a role to question and reflect upon what they saw and heard [61,62].

This approach was appropriate for the capture of ‘changing’ allowing studies of the dynamic *contexts* (e.g. political, institutional, economic, media, professional) within which the NHS CRS was being constructed, the varied processes that occurred in *making it work* (e.g. training, changing work practices, adapting and appropriating particular technologies, acts of improvisation and workarounds, negotiating shifting professional jurisdictions) and their implications. We can and do refer to these as *outcomes,* but of a rather different character to those associated with traditional evaluation. Our outcomes embody accounts of expectations, senses of benefits and risks, perceptions of consequences and processes of learning [44]. This fundamentally interpretive approach provided a set of rich accounts of local implementations which, when combined across sites, led to the production of generic insights, albeit not the conventional generalizations, concerning the different approaches to the implementation of national EHR initiatives and the sense of achievements attained [63].

### **Building the house: implications of our experience for other national EHR evaluations**

Any evaluations of large-scale or national EHR initiatives are likely to take place within changing political, economic and technical contexts, which will shape both the EHR and the evaluation in significant ways. Our moves from a comparative before-after methodology towards a case study methodology and an interpretive approach certainly changed the initial plans. We recognize too that these moves also limited the evaluation’s scope. For instance, we initially set out with the idea that our evaluation would give some weight to assessment of the impact of the NHS CRS on one of the conventional driving forces for EHR: error, safety and quality of care. However, the direct functionalities that these depended on were mostly unimplemented in the sites we studied within our timeframe. In terms of quality of care, only the availability of medical records in outpatient clinics was actually pursued in some sites [64].

But, while the lack of a before-after comparative analysis to support or refute claims for safety and quality is a limitation, and it will be very beneficial when and if multi-site EHR studies addressing these outcomes can be achieved, we take the inability to do this in our evaluation to be an inextricable part of real-time evaluation of any technology-led initiative in heathcare [65]. Beyond this, we see it as fundamental to understanding health IT that we *should* study carefully periods of change and changing. This type of evaluation will inevitably be contingent and hence we emphasize the need for reflexivity among researchers and adaptation of perspectives as have other authors writing on similar studies [66].

Some of our evaluation findings may not have a particularly direct relevance for other programs and settings with their own challenges and distinct characteristics. Indeed, the dimensions we have described here were to some degree outcomes of the value we (as researchers) attributed to (or inscribed into) EHR systems [67], depending on each researcher’s background, expertise and beliefs. This evaluation is thus like any other the result of processes of reduction, simplification and interpretation [47] and fundamentally subjective and a ‘partial truth’ [68].

The mainstream position in health services research would probably raise some objections to the position presented above. Thus, it has been argued that case study approaches lead to context-specific conclusions which hinder generalizability or transferability of findings across sites, let alone across countries [69]. But a statistical or positivist sense of generalizability is not, we argue, appropriate or meaningful here. Rather, in this work we adopted the interpretive position that trades off notions of sampling, significance and generalizability in favor of depth of enquiry, contextualization, abstraction, dialogue and critical reflection [30,60,70].

Our approach worked. We provided formative feedback to a number of Trusts and to NHS CFH at the time that it was most needed. The outputs of our research in the form of individual and collective case studies [64,71], provided rich accounts of how the NHS CRS (and more generally, the various manifestations of EHR systems) were understood and appropriated in a range of sites and by diverse professional interests. This constitutes a detailed stock of knowledge that can inform policy makers and managers at all levels, support learning within healthcare organizations, and provide transferable insights of relevance beyond the focus of our evaluation [72,73].

We are not, however, advocating interpretive process-focused studies as the sole or uniquely appropriate methodology for evaluating large-scale EHR programs and similar strategic interventions. But we do argue that in-situ and real-time evaluations benefit from an interpretive perspective focused on process if they are to inform policy at the time that it most needed. The interpretive approach in particular allows researchers to comprehend and reflect on the context and the means by which implementation and change occurs or not. Positivist approaches, including RCTs and before-after studies, despite health care’s long tradition and continuing calls for their use [11,19], are less able to conduct meaningful real-time formative evaluations that address such concerns. Despite the exalted status of RCTs as the gold standard for assessing cause and effect or therapeutic impact, they have only infrequently been used in EHR evaluations [19]. This has been due to multiple concerns including: the ethics of such approaches; difficulty to ‘control for’ potentially important effect mediators; associated costs and time-span [17,74]; impracticalities of randomizing parts of a hospital system; and difficulties in measuring the effects across a diverse array of outcomes [13,75,76].

We also see interpretive case-based evaluation as a useful way to avoid inherent pro-innovation bias, typically embedded in before-after evaluations [1,36,77]. Encouraging research respondents to narrate their own stories drawing from their past experiences, present understandings and future projections, builds a rich picture of the many processes that are at work, not all of which align directly with the innovation or technology in question. In presenting analysis and results, the use of a socio-technical language focused on practice can help us to see technology-led programs of change as being contingent and malleable – something in the making or being built – rather than something essential and fixed with defined and time-invariant causal properties [78].

Our sociotechnical lens was in particular focused on the specific question of how things were ‘made-to-work’ rather than on how well or not the EHR systems functioned [51]. By exploring EHR systems in-the-making, we were able to investigate and report on things that are of real concern to real policy makers and health systems managers – the causal texture of the domain within which implementation, adoption and use of innovations such as EHR take place. We were able to bring to the fore the intricate set of interlocking changes in practice that an EHR implies, a more formative view than the image of discrete even radical change that is the language of broad policy prescription and of technology vendors. From this perspective, non- or partial adoption, mis-use, non-use and workarounds, are not simply negative effects, pathologies or signs of failure, but are different enactments of the ‘technology-in-use’ [58,79]. Over a period of time they may chart the necessary path to a successful national EHR service.

### **Summary**

This paper reflects experience undertaking a real-time evaluation of a major program of EHR implementation in England. Many other countries have equivalent national plans or programs in various forms to facilitate clinical practice by the electronic capture and exchange of healthcare information. These will give rise to more large-scale evaluations. We suggest that in such cases, an outcome-centric evaluation approach is not in itself and alone adequate. To support this contention we have made two core arguments.

First, national EHR implementations take place within a complex, active and changing sociotechnical context. They engage with multiple stakeholders who have different understandings of EHR and of its potential purposes and different strategies for accommodating it (or not). The strong influence of the context means that technical elements of the EHR (i.e. the software, interface, database, and network) will require configuration or even substantial re-design to meet local needs and interests and to match extant business processes. In other words, the core technology and its accompanying message of a particular type of change is not strong enough to lay down or enforce a definitive account of what EHR will become. Just as national EHR programs cannot be dissociated from the national social and political context, neither can they be disassociated from the local contexts, the place where they come engaged with managerial and clinical practice and changing occurs. This suggests that using the presence or absence of EHR technology as the fundamental basis of evaluation research design (before-after) is misleading and inadequate. This leads to the second argument, made in this paper. Conventional evaluations such as those employing RCT or before-after designs, despite forming the bedrock of Evidence Based Medicine, are not capable of giving essential insight into the nature and degree of transformative power that national EHR initiatives may have. Hence the need for methodological reflexivity and adaptation (often radical), including a willingness to embrace new ontological, epistemological and methodological assumptions.

This position is not, in truth, so far from the experience of those who work day-to-day with technology or manage healthcare organizations. Policy makers, implementers and other stakeholders are not naïve and seldom perceive implementation as just a linear process that starts off with finding the ‘right’ technology and putting it in place so that the intended users use it as planned with the expected outcomes being achieved. Rather, it is understood, there are tensions among significant stakeholders as they balance parallel assumptions and expectations about technology and about their work. For these reasons we suggest there is no single, or standard way of best implementing national and large-scale EHR systems, and so too there is no predefined and prescriptive strategy to evaluate them. Thus our intention is not to advocate an interpretive methodology as against a positivist methodology *per se*, but to highlight what can be achieved through the practice of methodological reflexivity and adaptability.

The shifting sands are unavoidable, but we must nonetheless set to work *building* EHR houses that we can then all comfortably live in.

## **Endnotes**

^1^ Although there is no universally agreed definition of EHR [1], in the context of this paper we use the term to refer to a digital, longitudinal patient record that is available to healthcare providers across a range of clinical settings [8].

^2^ Each NHS Trust comprises one or more hospitals and/or NHS services, such as acute care, community or mental health services. For ease of reading we sometimes use the term ‘hospital’ in this paper, and sometimes Trust.

^3^http://www.haps2.bham.ac.uk/publichealth/cfhep/research.shtml

## Competing interests

The authors declare that they have no competing interests.

## **Authors’ contributions**

AT and DP drafted the first versions of the manuscript with TC, AS and NB, who all extensively contributed to several revisions and intellectual development of the article. All authors read and approved the final manuscript.

## **Funding**

This report is independent research commissioned by the National Institute for Health Research. The views expressed in this publication are those of the authors and not necessarily those of the NHS, the National Institute for Health Research or the Department of Health.

## Pre-publication history

The pre-publication history for this paper can be accessed here:

http://www.biomedcentral.com/1472-6963/12/105/prepub
